# Modulating the Chemical and Biological Properties of Cancer Stem Cell-Potent Copper(II)-Nonsteroidal Anti-Inflammatory Drug Complexes

**DOI:** 10.3390/molecules24091677

**Published:** 2019-04-29

**Authors:** Jimin Shin, Arvin Eskandari, Kogularamanan Suntharalingam

**Affiliations:** Department of Chemistry, King’s College London, London SE1 1DB, UK; jimin.shin@kcl.ac.uk (J.S.); arvin.eskandari@kcl.ac.uk (A.E.)

**Keywords:** metallodrugs, bioinorganic chemistry, copper, cancer stem cells

## Abstract

Copper(II) complexes bearing nonsteroidal anti-inflammatory drugs (NSAIDs) are known to potently kill cancer stem cells (CSCs), a subpopulation of tumour cells with high metastatic and relapse fidelity. One of the major disadvantages associated to these copper(II) complexes is their instability in the presence of strong cellular reductants (such as ascorbic acid). Here we present a biologically stable copper(II)-NSAID complex containing a bathocuproinedisulfonic acid disodium ligand and two indomethacin moieties, Cu(bathocuproinedisulfonic acid disodium)(indomethacin)_2_, **2**. The copper(II) complex, **2** kills bulk breast cancer cells and breast CSC equally (in the sub-micromolar range) and displays very low toxicity against non-tumorigenic breast and kidney cells (IC_50_ value > 100 µM). Three-dimensional cell culture studies show that **2** can significantly reduce the number and size of breast CSC mammospheres formed (from single suspensions) to a similar level as salinomycin (an established anti-breast CSC agent). The copper(II) complex, **2** is taken up reasonably by breast CSCs and localises largely in the cytoplasm (>90%). Cytotoxicity studies in the presence of specific inhibitors suggest that **2** induces CSC death via a reactive oxygen species (ROS) and cyclooxygenase isoenzyme-2 (COX-2) dependent apoptosis pathway.

## 1. Introduction

Cancer is one of the leading causes of death worldwide [1]. Advancements in treatments (including chemotherapy, radiotherapy, surgery, and immunotherapy) have significantly improved long-term survival rates and the quality of life for patient suffering from various cancer types [1]. Platinum(II)-containing compounds such as cisplatin, carboplatin, and oxaliplatin are a widely used class of chemotherapeutic drugs—it is believed that up to 50% of all cancer patients are treated with one of these platinum(II) complexes at some point during their therapeutic regimen [2,3]. Despite the huge success of these platinum agents, they are plagued with drawbacks such as non-specificity (leading to systemic toxicity and side-effects), susceptibility to acquired resistance (leading to ineffective treatment against several cancer types), and the inability to prevent reoccurrence (leading to relapse with fatal consequences) [4,5]. The existence of cancer stem cells (CSCs), a sub-population of tumour cells with the ability to differentiate, self-renew, and seed secondary tumours, is thought to contribute to cancer relapse [6,7]. The anticancer platinum drugs, like all other clinically approved chemotherapeutics, are unable to remove CSCs at their therapeutically administered doses [8,9,10,11]. Therefore, there is a need for the development of new chemotherapeutics, including those containing metals, that can remove CSCs at clinically safe doses and thus potentially overcome cancer relapse. Over the last few years we and others have developed several novel metal complexes with potent CSC activity [12].

**Figure 1:**
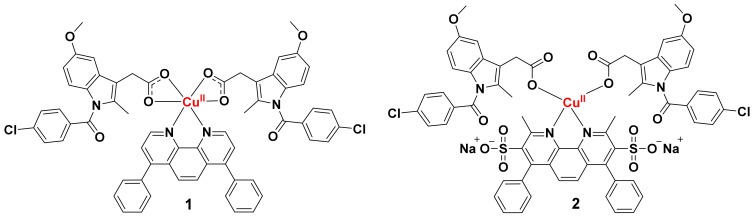
Chemical structures of copper(II) complexes containing a 4,7-diphenyl-1,10-phenanthroline unit and two indomethacin ligands under investigation in this study. The six-coordinate complex, **1** was previously shown, by us, to potently and selectivity kill breast cancer stem cells (CSCs) (over bulk breast cancer cells). The proposed four-coordinate complex, **2** is envisaged to be more stable than **1** towards biological reduction.

One of the most promising classes of anti-CSCs agents identified thus far is copper(II)-phenanthroline complexes bearing nonsteroidal anti-inflammatory drugs (NSAIDs) [13,14]. It should be noted that several metal-NSAIDs complexes have been developed and shown to exhibit attractive antibacterial and antiproliferative properties [15]. Within this family of compounds, a copper(II)-4,7-diphenyl-1,10-phenanthroline complex bearing two indomethacin moieties, Cu(bathophenanthroline)(indomethacin)_2_, **1** (attached to the copper centre via a bidentate carboxylate interaction as depicted in Figure 1) was found to be the most promising candidate, with potent (sub-micromolar) and selective (3-fold over breast bulk cancer cells) toxicity towards breast CSCs. Mechanistic studies revealed that **1** induces breast CSC death by increasing intracellular reactive oxygen species (ROS) levels to lethal doses and inhibiting cyclooxygenase isoenzyme (COX-2). COX-2 is overexpressed in breast CSCs and involved in its proliferation and maintenance [16,17]. Recent biophysical studies showed that **1** disassembles in presence of biological reductants (such as ascorbic acid). Specifically, the complex sheds both the indomethacin and 4,7-diphenyl-1,10-phenanthroline ligands and forms a copper(I) species [18]. Degradation of **1** upon biological reduction arises partly due to the difference in geometrical preference of the oxidized and reduced forms. The copper(II), d^9^ centre in **1** (oxidized form) adopts a six-coordinate (distorted octahedral) geometry whereas the copper(I), d^10^ centre in reduced **1** is likely to favour a four-coordinate (distorted tetrahedral) geometry. Here we have sought to improve the stability of **1**, by preparing a four-coordinate Cu(bathocuproinedisulfonic acid disodium)(indomethacin)_2_ complex, **2** that upon reduction to the copper(I) form has a high likelihood of remaining intact because the geometrical reorganisation energy required for reduction is relatively small (as both the oxidized and reduced forms of **2** are likely to adopt a four-coordinate system). To facilitate the formation of this relatively unusual, four-coordinate copper(II) complex with a 4,7-diphenyl-1,10-phenanthroline motif and two indomethacin units, the bathocuproinedisulfonic acid disodium salt was used. The methyl groups on the 2- and 9-positions of the bathocuproinedisulfonic acid disodium salt prevent the formation a copper(II) six-coordinate system with indomethacin, due to steric hindrance. Instead a four-coordinate complex is envisaged with the copper(II) centre bound to the bathocuproinedisulfonic acid disodium moiety via two nitrogen atoms and to two indomethacin units via monodentate interactions (as depicted in Figure 1). Indeed, copper(II) complexes with two 2,9-dimethyl-1,10-phenanthroline ligands, or one 2,9-dimethyl-1,10-phenanthroline ligand and two monodentate ligands have been reported to adopt distorted tetrahedral structures [19,20,21]. The presence of the disulfonic acid disodium groups promotes water solubility, therefore as well as being more stable towards biological reduction (than **1**), **2** is expected to be more hydrophilic (than **1**) and thus more suitable for in vivo administration and drug formulation. As well as reporting the synthesis and characterization of **2**, we present the cytotoxicity of **2** against bulk cancer cells, breast CSCs, non-tumorigenic cells and its mechanism of cytotoxicity in breast CSCs. 

## 2. Results and Discussion

The six-coordinate copper(II) complex, **1** was prepared using an established protocol previously reported by us [14]. The novel copper(II) complex, **2** was prepared by reacting CuCl_2_·2H_2_O with bathocuproinedisulfonic acid disodium salt and two equivalents of indomethacin in methanol:water (30:1), in the presence of excess KOH, open to the air (see Scheme S1). The copper(II) complex, **2** was isolated as a green solid and characterised by mass spectrometry, infra-red spectroscopy, and elemental analysis (see Supporting Information). A molecular ion peak corresponding to intact **2** (as the potassium salt) was observed in the positive mode of the ESI mass spectrum (*m/z* = 1371.8762, [potassium salt **2**-H]^+^) (Figure S1). The elemental composition report for the assigned molecular ion peak matches the predicted molecular formula for the potassium salt of **2**. For free indomethacin, the vibrational stretching frequencies, ν(C=O) and ν(C-O) associated to the carboxylic acid moiety, appear at 1716 and 1290 cm^−1^ respectively (Figure S2). Upon binding to a metal, the difference between the vibrational stretching frequencies between the asymmetric, ν_asym_(CO_2_) and symmetric, ν_sym_(CO_2_) carboxylato group peaks gives an indication into the binding mode of the carboxylato group to the metal centre [22,23]. Therefore careful IR analysis allowed us to determine the binding mode of the two indomethacin ligands in **2** to the copper(II) centre. According to the IR spectrum of **2**, the difference (Δ) between the ν_asym_(CO_2_) and ν_sym_(CO_2_) stretching bands varied by 238 cm^−1^ (Figure S3), suggestive of a monodentate binding mode for the carboxylate group on indomethacin to the copper(II) centre (as depicted in Figure 1). This means that **2** is, most likely, a four-coordinate complex and not a six-coordinate complex like, previously reported, for **1**. The ^1^H NMR spectrum of **2** in DMSO-d_6_ displayed broad peaks that could be tentatively assigned to protons on the indomethacin and bathocuproinedisulfonic acid disodium moieties (tentatively assignments of the broad and often coalesced peaks are provided in Figure S4). The ^1^H NMR spectrum of indomethacin in DMSO-d_6_ was recorded for comparison (Figure S5). The broad nature of the signals for **2** suggests that the copper atom in **2** is in the paramagnetic, copper(II), d^9^ form and not the diamagnetic, copper(I), d^10^ form. The high purity and chemical composition of **2** was confirmed by elemental analysis.

**Figure 2:**
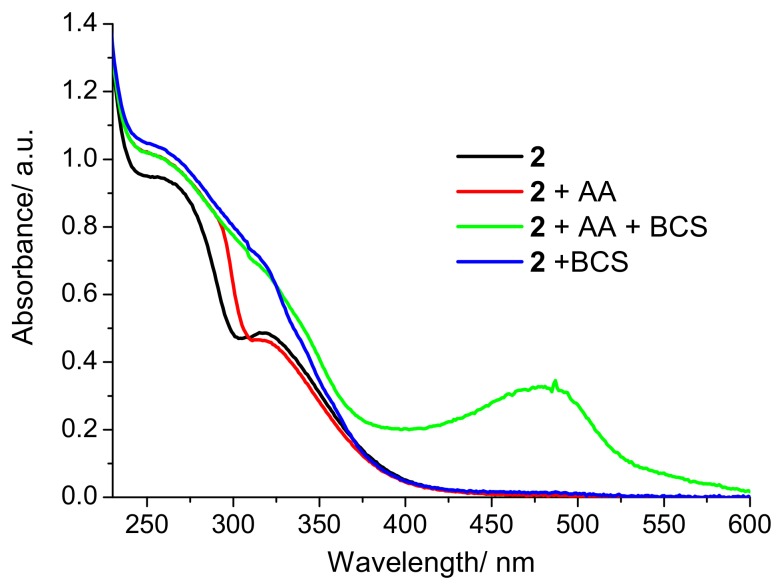
UV-Vis spectrum of **2** (50 μM) in PBS:DMSO (200:1) (black line), **2** (50 μM) in the presence of ascorbic acid (500 μM) in PBS:DMSO (200:1) (red line), **2** (50 μM) in the presence of ascorbic acid (500 μM) and bathocuproine disulfonate, BCS (100 μM) in PBS:DMSO (200:1) (green line), **2** (50 μM) in the presence of bathocuproine disulfonate, BCS (100 μM) in PBS:DMSO (200:1) (blue line), all at 37 °C.

**Scheme 1:**
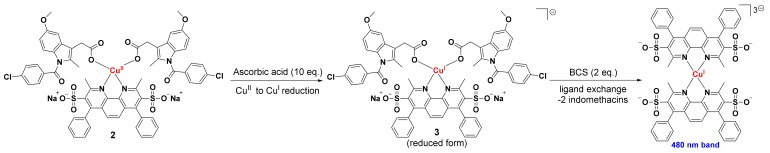
Proposed reaction scheme for the reduction of **2** by ascorbic acid (10 eq.) and subsequent reaction with bathocuproinedisulfonic acid disodium in PBS:DMSO (200:1) at 37 °C.

UV-Vis spectroscopy studies were carried out to assess the chemical integrity of **2** in biologically relevant solutions. In PBS:DMSO (200:1), **2** (50 µM) was completely stable over a period of 24 h at 37 °C (Figure S6). In the presence of ascorbic acid (10 equivalents), the absorption of **2** (50 µM) remained largely unaltered over the course of 24 h at 37 °C (Figure S7), indicative of stability. The low energy band at 320 nm corresponding to metal-perturbed π-π* transitions associated to the indomethacin and bathocuproinedisulfonic acid disodium ligands was relatively unaffected, implying that the geometry of **2** did not change significantly after reduction (by ascorbic acid). These results are in stark contrast to those previously reported for **1** under identical conditions. In the presence of ascorbic acid (10 equivalents) in PBS:DMSO (200:1), the absorption of **1** (50 µM) changed dramatically over the course of 24 h at 37 °C, suggestive of instability [18]. Detailed biophysical studies showed that **1** liberated both the indomethacin and 4,7-diphenyl-1,10-phenanthroline ligands upon reduction by ascorbic acid [18]. To show that **2** is reduced by ascorbic acid, additional UV-Vis spectroscopy studies were carried with excess bathocuproinedisulfonic acid disodium (two equivalents), a strong copper(I) chelator [24]. Upon addition of bathocuproinedisulfonic acid disodium (two equivalents) to a PBS:DMSO (200:1) solution of **2** (50 µM) and ascorbic acid (10 equivalents), a characteristic absorption band at 480 nm corresponding to [Cu^I^(BCS)_2_]^3−^ was observed (Figure 2). The formation of [Cu^I^(BCS)_2_]^3−^ under these conditions is likely to results from the reduction of **2** to the copper(I) form, **3** (by ascorbic acid) and subsequent displacement of the indomethacin ligands by bathocuproinedisulfonic acid disodium (as depicted in Scheme 1). The addition of bathocuproinedisulfonic acid disodium to **2** (50 µM) in the absence of ascorbic acid did not produce an absorption band at 480 nm, implying that **2** must be reduced to the copper(I) form before displacement of the indomethacin ligands can occur (Figure 2 and Scheme 1). Taken together, the UV-Vis spectroscopy studies show that **2** is significantly more stable than **1** in biologically reducing conditions. More specifically, when **2** is reduced from the copper(II) to copper(I) form by ascorbic acid, it appears that its structural integrity as a four-coordinate complex is retained. 

Complete or partial solubility of biologically active compounds in aqueous solutions is vital for drug formulation and in vivo administration [25]. The lipophilicity of **2** was determined by measuring the extent to which it partitioned between octanol and water, P. The experimentally determined LogP values for **2** was 18% lower than that reported for **1** (0.83 for **2** and 1.01 for **1**) [14], proving **2** is more water soluble than **1**. This can be attributed to the ionic nature of the disulfonic acid disodium groups on the bathocuproinedisulfonic acid disodium ligand on **2**. Prior to preforming cellular studies, the stability of **2** in mammary epithelial cell growth medium (MEGM) supplemented with ascorbic acid (10 equivalents) at 37 °C was established (Figure S8).

The cytotoxicity of **2** against human bulk breast cancer/CSC-depleted cells (HMLER) and breast CSC-enriched cells (HMLER-shEcad) was determined using the MTT (3-(4,5-dimethylthiazol-2-yl)-2,5-diphenyltetrazolium bromide) assay. The IC_50_ values were determined from dose-response curves (Figure S9) and are summarized in Table 1. The copper(II) complex, **2** indiscriminately kills bulk breast cancer cells and breast CSCs in the sub-micromolar range. Therefore, it has the potential to remove whole breast tumour populations (including bulk cancer cells and CSCs) with a single dose. Notably, **2** displaces 19-fold higher potency toward CSC-enriched HMLER-shEcad cells than salinomycin, an established anti-CSC agent currently undergoing clinical development [13,26]. The IC_50_ values of **2** towards HMLER and HMLER-shEcad cells are similar to **1** [14], showing that replacement of 4,7-diphenyl-1,10-phenanthroline in **1** with bathocuproinedisulfonic acid disodium in **2** and the subsequent change in coordination number, does not detrimentally affect potency towards breast cancer cells (bulk cancer cells and CSCs). We have previously shown that indomethacin is non-toxic towards HMLER and HMLER-shEcad cells (IC_50_ value > 100 µM) [13], therefore the potency of **2** is likely to originate from the copper(II)-bathocuproinedisulfonic acid disodium component. The toxicity of **2** toward non-tumorigenic cells (human embryonic kidney HEK 293T cells and human epithelial breast MCF710A cells) was also determined using the MTT assay (Figure S9). Strikingly, **2** was non-toxic towards HEK 293T and MCF10A cells (IC_50_ value > 100 µM). Collectively, the cytotoxicity data suggest **2** can potentially remove breast cancer cell populations in their entirety (bulk cancer cells and CSCs) with a single dose, with reduced toxicity towards non-cancerous cells.

**Figure 3:**
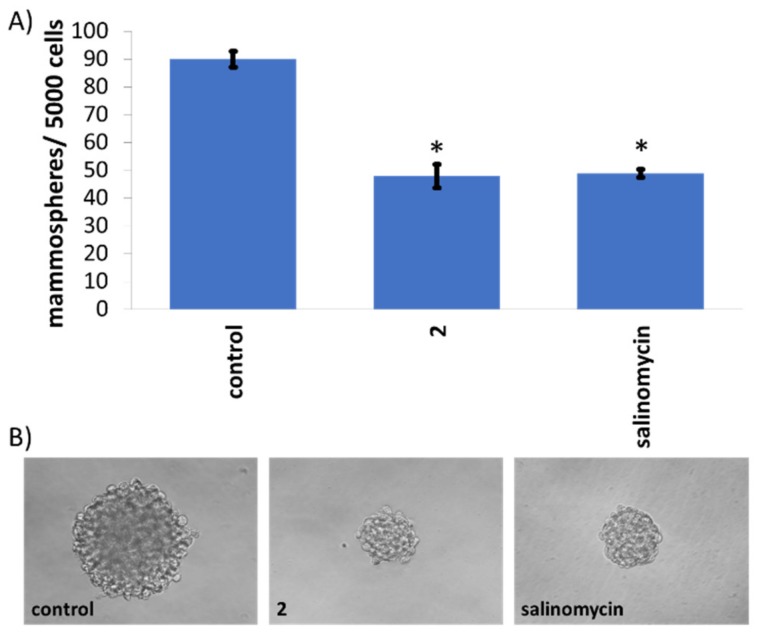
(**A**) Quantification of mammosphere formation with HMLER-shEcad cells untreated and treated with **2** and salinomycin at their respective IC_20_ values for five days. Error bars = SD and Student’s *t*-test, *= *p* < 0.05. (**B**) Representative images (×20) of HMLER-shEcad mammospheres in the absence (control) and presence of **2** and salinomycin at their respective IC_20_ values for five days.

To provide more clinically relevant potency data, mammosphere studies were performed. When CSCs are cultured in serum-free media, under low-attachment conditions, spheriods representative of tumours are formed. These structures provide a reliable in vitro model to probe CSC activity. The ability of **2** to inhibit HMLER-shEcad mammosphere formation from single cell suspensions at non-lethal doses was determined using an inverted microscope. Treatment with **2** (at the IC_20_ value after five days incubation) significantly reduced (*p* < 0.05) the number and size of mammospheres formed (Figure 3A,B). A similar result was observed for salinomycin (Figure 3A,B), and previously for **1** [14]. This shows that despite the structural differences between **1** and **2**, the mammosphere inhibitory properties of **2** is not compromised. 

**Figure 4:**
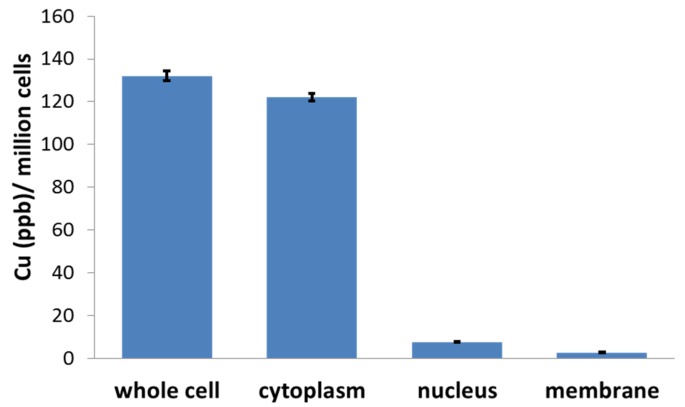
Copper content in whole cell, cytoplasm, nucleus and membrane fractions isolated from HMLER-shEcad cells treated with **2** (1 µM for 16 h).

Cellular uptake studies were performed to determine the CSC permeability of **2**. HMLER-shEcad cells were incubated with **2** (1 µM for 16 h) and the intracellular copper content was determined by inductively coupled plasma mass spectrometry (ICP-MS). The complex, **2** was reasonably internalised by CSCs, with whole cell uptake totalling 132 ± 2 ppb of Cu/million cells (Figure 4 and Figure S8). The CSC uptake of **1** under identical conditions (1 µM for 16 h) was 4-fold higher (522 ± 5 ppb of Cu/million cells) than **2** (Figure S10). This is consistent with their respective LogP values i.e., **1** is more lipophilic than **2** and thus predicted to internalise better. Fractionation studies showed that 92% (122 ± 2 ppb of Cu/million cells) of internalised **2** was detected in the cytoplasm (Figure 4). The remainder (8%) of internalised **2** was found in the nucleus and membrane (Figure 4). This suggests that the CSC potency of **2** is likely to be due to damage to cyctoplasmic proteins or other cyctoplasmic biological components.

The parent copper(II)-NSAID complex, **1** induces CSC apoptosis by increasing ROS levels and inhibiting COX-2. To determine if **2** evokes a similar CSC response to **1**, cytotoxicity studies were carried in the presence of specific inhibitors. The IC_50_ value of **2** towards CSC-enriched HMLER-shEcad cells significantly (*p* > 0.05) increased in the presence of *N*-acetylcysteine (2 mM, an established ROS scavenger, Figure S11) to 0.57 ± 0.01 µM, prostaglandin E2 (PGE2) (20 µM, the functional product of COX-2-catalysed arachidonic acid metabolism, Figure S11) to 0.93 ± 0.03 µM, and z-VAD-FMK (5 µM, a peptide-based apoptosis inhibitor, Figure S11) to 0.76 ± 0.04 µM. This suggests that like **1**, **2**-induces CSC death via a ROS and COX-2 dependent apoptosis pathway.

In summary we report the synthesis, characterisation, and anti-CSC properties of a copper(II) complex comprising of a bathocuproinedisulfonic acid disodium ligand and two indomethacin units, **2**. Unlike previously reported CSC-potent copper(II)-NSAID complexes, **2** is stable under biologically reducing conditions. The copper(II) complex, **2** potently kills bulk breast cancer cells and breast CSCs. Remarkably, **2** displays very low toxicity towards healthy breast and kidney cells. The complex, **2** is also able to inhibit the formation of three-dimensional breast CSC mammospheres to a similar extent as salinomycin. Mechanistic studies suggest that **2** induces breast CSC death by apoptosis as a consequence of intracellular ROS generation and COX-2 inhibition. Overall we show that by influencing the geometry of CSC-potent copper(II)-NSAID complexes, through the choice of coordinating ligand(s), biologically stable analogues can be developed without loss of CSC potency. 

## 3. Experimental Details

### 3.1. Materials and Methods

All synthetic procedures were performed under normal atmospheric conditions. Fourier transform infrared (FTIR) spectra were recorded with an IRAffinity-1S Shimadzu spectrophotometer. High resolution electron spray ionisation mass spectra were recorded on a BrukerDaltronics Esquire 3000 spectrometer by Dr. Lisa Haigh (Imperial College London). UV-Vis absorption spectra were recorded on a Cary100 UV-Vis spectrophotometer. ^1^H NMR spectra were recorded on a BrukerAvance 400 MHz Ultrashield NMR spectrometer. ^1^H NMR spectra were referenced internally to residual solvent peaks, and chemical shifts are expressed relative to tetramethylsilane, SiMe_4_ (δ = 0 ppm). Elemental analysis of the compounds prepared was performed commercially by London Metropolitan University. The Cu(4,7-diphenyl-1,10-phenanthroline)(indomethacin)_2_ complex, **1** was prepared according to a previously reported protocol [14]. Bathocuproinedisulfonic acid disodium salt, 4,7-diphenyl-1,10-phenanthroline, and indomethacin were purchased from Sigma Aldrich and used as received. 

*Synthesis of Cu(Bathocuproinedisulfonic acid disodium)(Indomethacin)*_2_ (**2**). Indomethacin (151 mg, 0.42 mmol) and KOH (29.7 mg, 0.53 mmol) were stirred in methanol (5 mL) for 1 h. The indomethacin/KOH methalonic solution and a methalonic solution (5 mL) of the bathocuproinedisulfonic acid disodium salt (121 mg, 0.21 mmol) were added simultaneously and dropwise to another methanol:water (10:1) solution (5 mL) of CuCl_2_ (31 mg, 0.23 mmol). The mixture was heated at 50 °C for 20 h. The resultant precipitate was filtered off and washed thoroughly with water (3 × 20 mL) and diethyl ether (3 × 20 mL) to yield **2** as a green solid (43 mg, 15%); IR (solid, cm^−1^): 1654, 1638, 1606, 1580, 1506, 1453, 1400, 1368, 1272, 1257, 1235, 1214, 1161, 1034, 928, 896, 859, 753, 705, 668, 567; ESI-MS Calcd. for C_64_H_47_C_l2_CuK_2_N_4_O_14_S_2_ [potassium salt **2-**H]^+^: 1371.9008 a.m.u. Found [potassium salt **2-**H]^+^: 1371.8762 a.m.u; Anal. Calcd. for **2**, C_64_H_48_C_l2_CuN_4_Na_2_O_14_S_2_: C, 57.28; H, 3.61; N, 4.18. Found: C, 57.20; H, 3.72; N, 4.05.

### 3.2. Measurement of Water-Octanol Partition Coefficient (Log P)

The LogP value for **2** was determined using the shake-flask method and UV-Vis spectroscopy. The octanol used in this experiment was pre-saturated with water. An aqueous solution of **2** (500 μL, 100 μM) was incubated with octanol (500 μL) in a 1.5 mL tube. The tube was shaken at room temperature for 24 h. The two phases were separated by centrifugation and **2** content in each phase was determined by UV-Vis spectroscopy.

### 3.3. Cell Lines and Cell Culture Conditions

The human embryonic kidney HEK 293T cell line was acquired from the American Type Culture Collection (ATCC, Manassas, VA, USA) and maintained in Dulbecco’s Modified Eagle’s Medium (DMEM) supplemented with 10% fetal bovine serum and 1% penicillin/streptomycin. The human mammary epithelial cell lines, HMLER and HMLER-shEcad were kindly donated by Prof. R. A. Weinberg (Whitehead Institute, MIT). The human epithelial breast MCF710A cell line was acquired from the American Type Culture Collection (ATCC, Manassas, VA, USA). HMLER, HMLER-shEcad, and MCF10A cells were maintained in Mammary Epithelial Cell Growth Medium (MEGM) with supplements and growth factors (BPE, hydrocortisone, hEGF, insulin, and gentamicin/amphotericin-B). The cells were grown at 310 K in a humidified atmosphere containing 5% CO_2_.

### 3.4. Cytotoxicity MTT Assay

The colourimetric MTT assay was used to determine the toxicity of **2**. HMLER, HMLER-shEcad, MCF10A, or HEK 293T cells (5 × 10^3^) were seeded in each well of a 96-well plate. After incubating the cells overnight, various concentrations of the compounds (0.2–100 µM), were added and incubated for 72 h (total volume 200 µL). Stock solutions of the compounds were prepared as 10 mM solutions in DMSO and diluted using media. The final concentration of DMSO in each well was 0.5% and this amount was present in the untreated control as well. After 72 h, 20 μL of a 4 mg/mL solution of MTT in PBS was added to each well, and the plate was incubated for an additional 4 h. The MEGM/MTT or DMEM/MTT mixture was aspirated and 200 μL of DMSO was added to dissolve the resulting purple formazan crystals. The absorbance of the solutions in each well was read at 550 nm. Absorbance values were normalized to (DMSO-containing) control wells and plotted as concentration of test compound versus % cell viability. IC_50_ values were interpolated from the resulting dose dependent curves. The reported IC_50_ values are the average of three independent experiments, each consisting of six replicates per concentration level (overall n = 18).

### 3.5. Tumorsphere Formation and Viability Assay 

HMLER-shEcad cells (5 × 10^3^) were plated in ultralow-attachment 96-well plates (Corning) and incubated in MEGM supplemented with B27 (Invitrogen), 20 ng/mL EGF, and 4 µg/mL heparin (Sigma) for 5 days. Studies were conducted in the absence and presence of **2** and salinomycin. Mammospheres treated with **2** and salinomycin (at their respective IC_20_ values, 5 days) were counted and imaged using an inverted microscope. 

### 3.6. Cellular Uptake

To measure the cellular uptake of **1** and **2** *ca.* 1 million HMLER-shEcad cells were treated with **1** and **2** (1 μM) at 37 °C for 16 h. After incubation, the media was removed, the cells were washed with PBS (2 mL × 3), harvested. The number of cells was counted at this stage, using a haemocytometer. This mitigates any cell death induced by **1** and **2** at the administered concentration and experimental cell loss. The cells were centrifuged to form pellets. For **2**, cellular pellets were also used to determine the copper content in the nuclear, cytoplasmic, and membrane fractions. The Thermo Scientific NE-PER Nuclear and Cytoplasmic Extraction Kit was used to extract and separate the nuclear, cytoplasmic, and membrane fractions. The cellular pellets were dissolved in 65% HNO_3_ (250 μL) overnight. All samples were diluted 5-7-fold with water and analysed using inductively coupled plasma mass spectrometry (ICP-MS, PerkinElmer NexION 350D). Copper levels are expressed as Cu (ppb) per million cells. Results are presented as the mean of five determinations for each data point. 

## Figures and Tables

**Table 1:** IC_50_ values of **1**, **2**, and salinomycin against human bulk breast cancer/CSC-depleted cells (HMLER) and breast CSC-enriched cells (HMLER-shEcad) cells determined after 72 h incubation (mean of three independent experiments ± SD).

Compound	HMLER IC_50_ (μM)	HMLER-shEcad IC_50_ (μM)
**1** ^1^	0.46 ± 0.16	0.23 ± 0.04
**2**	0.31 ± 0.01	0.22 ± 0.01
Salinomycin ^2^	11.4 ± 0.4	4.2 ± 0.3

^1^ Taken from [14]. ^2^ Taken from [13].

## Supplementary Materials

The following are available online, Experimental Details (including Figures S1–S11).
