# Supplementary material for: Modulating the Chemical and Biological Properties of Cancer Stem Cell-Potent Copper(II)-Nonsteroidal Anti-Inflammatory Drug Complexes
Source: Molecules. 2019 Apr 29;24(9):1677. doi: 10.3390/molecules24091677 (PMC6540347; doi:10.3390/molecules24091677)
Supplement: Supplementary file 1 [file molecules-24-01677-s001.pdf]

Supporting Information

Modulating the Chemical and Biological Properties of Cancer Stem Cell-Potent Copper(II)-Nonsteroidal Anti-Inflammatory Drug Complexes

Jimin Shin,[a] Arvin Eskandari,[a] and Kogularamanan Suntharalingam*[a]

[a] Department of Chemistry, King's College London, London, SE1 1DB, United Kingdom

Email: kogularamanan.suntharalingam@kcl.ac.uk

<u>Table of Content</u>

**Scheme S1.**	Chemical reaction scheme for the preparation of complex **2**.
**Figure S1.**	High resolution ESI mass spectrum (positive mode) of **2**.
**Figure S2.**	IR spectrum of indomethacin in the solid form.
**Figure S3.**	IR spectrum of **2** in the solid form.
**Figure S4.**	^{1}H NMR spectrum of **2** in DMSO-d$_6$.
**Figure S5.**	^{1}H NMR spectrum of indomethacin in DMSO-d$_6$.
**Figure S6.**	UV-Vis spectrum of **2** (50 μM) in PBS (pH 7.4)/DMSO (200:1) over the course of 24 h at 37 °C.
**Figure S7.**	UV-Vis spectrum of **2** (50 μM) in PBS (pH 7.4)/DMSO (200:1) in the presence of ascorbic acid (500 μM) over the course of 24 h at 37 °C.
**Figure S8.**	UV-Vis spectrum of **2** (50 μM) in mammary epithelial cell growth medium (MEGM):DMSO (200:1) in the presence of ascorbic acid (500 μM) over the course of 72 h at 37 °C.
**Figure S9.**	Representative dose-response curves for the treatment of HMLER, HMLER-shEcad, MCF10A, and HEK293T cells with **2** after 72 h incubation.
**Figure S10.**	Copper content in whole-cell isolated from HMLER-shEcad cells treated with **1** and **2** (1 μM for 24 h).
**Figure S11.**	Representative dose response curves of **2** against HMLER-shEcad cells in the absence and presence of *N*-acetylcysteine (2 mM), or PGE2 (20 μM), or z-VAD-FMK (5 μM) after 72 h incubation.

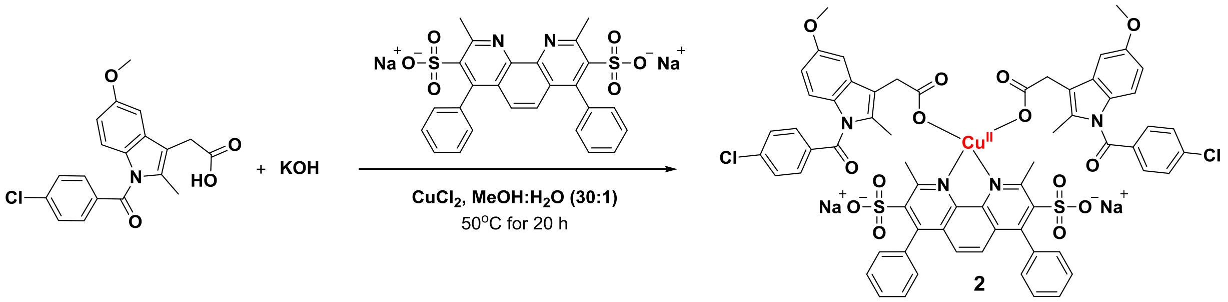

Scheme S1. Chemical reaction scheme for the preparation of complex **2**.

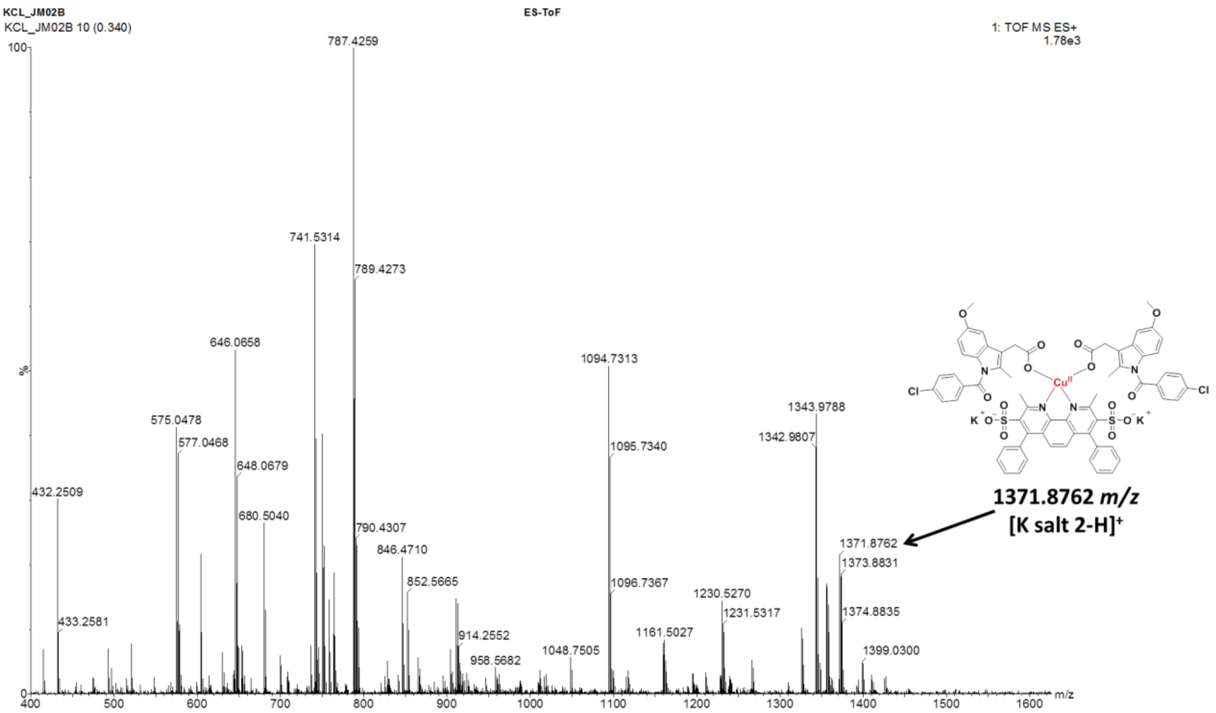

Figure S1. High resolution ESI mass spectrum (positive mode) of **2**. The elemental composition report for the assigned molecular ion peak matches the predicted molecular formula for the potassium salt of **2**.

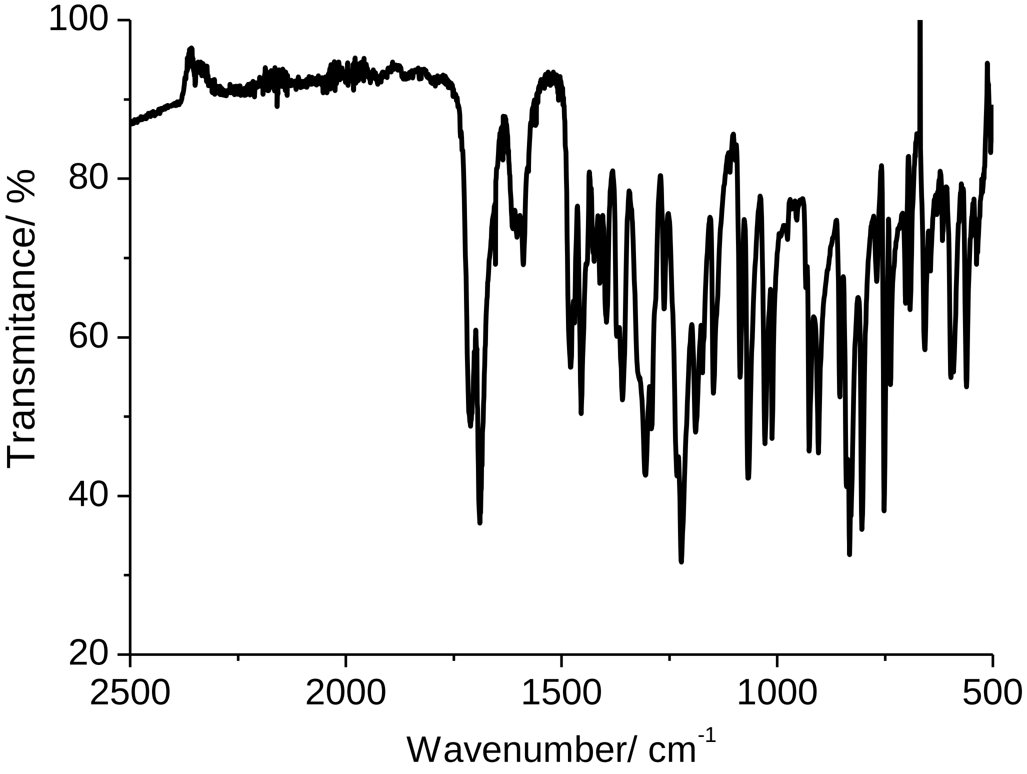

Figure S2. IR spectrum of indomethacin in the solid form.

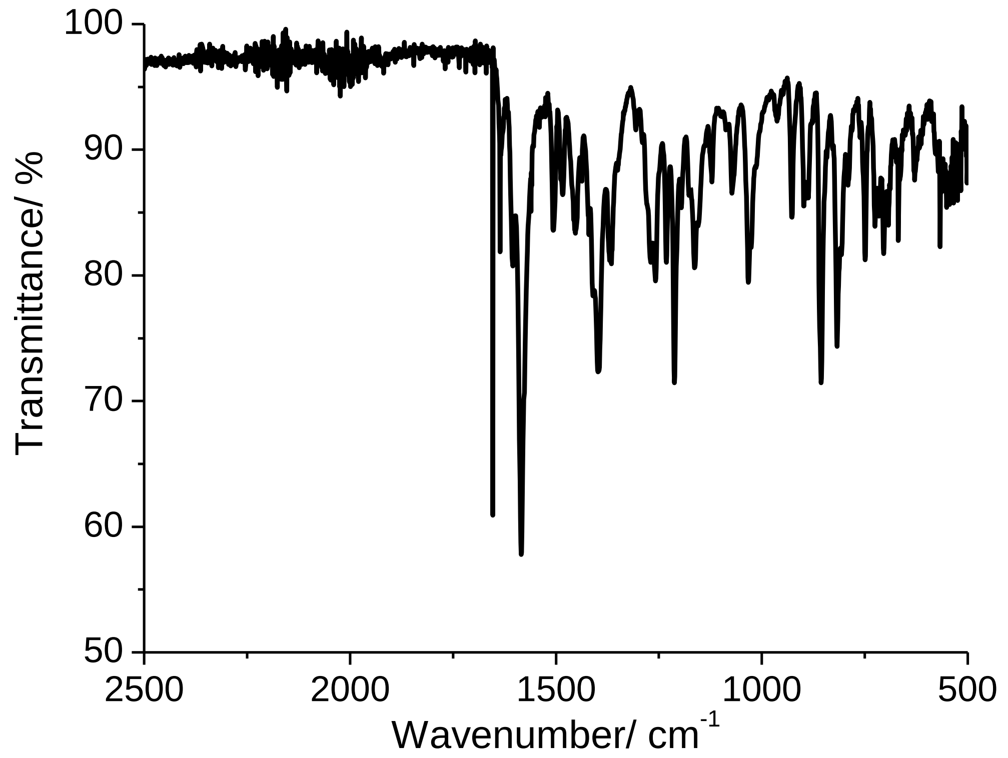

Figure S3. IR spectrum of **2** in the solid form.

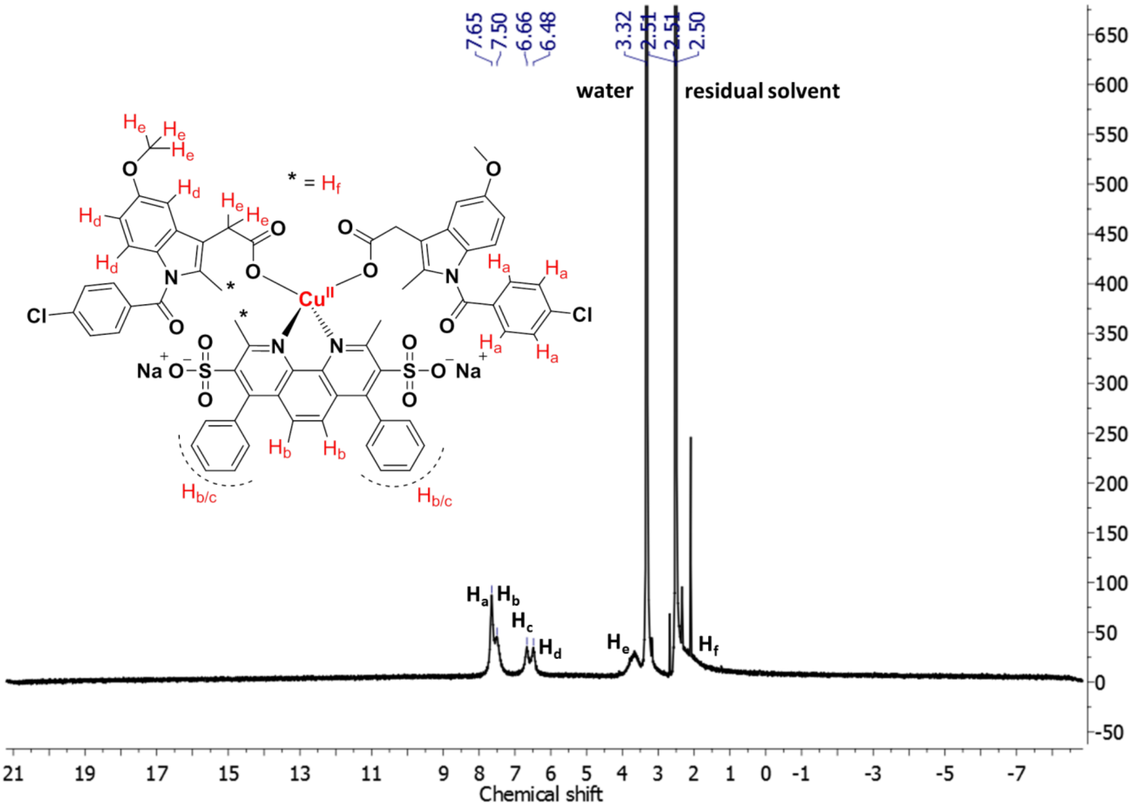

Figure S4. ^{1}H NMR spectrum of **2** in DMSO-d_6.

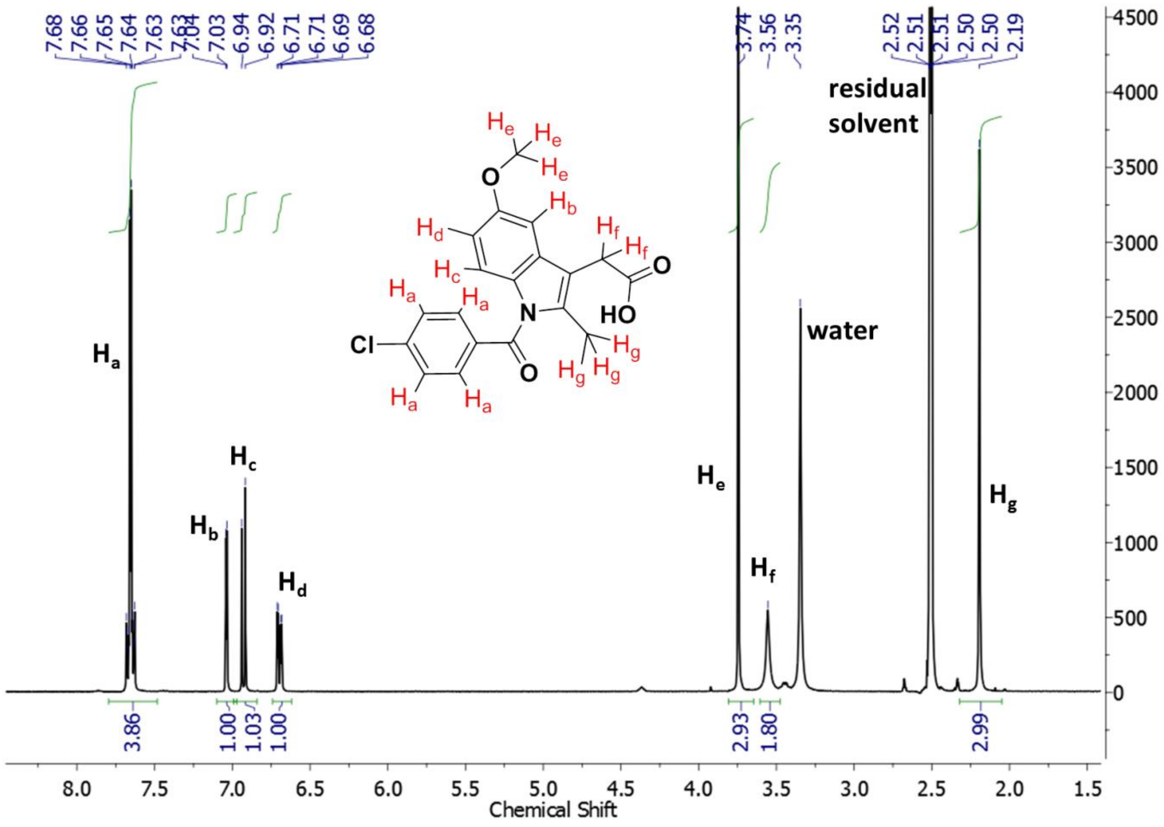

Figure S5. ^{1}H NMR spectrum of indomethacin in DMSO-d$_6$.

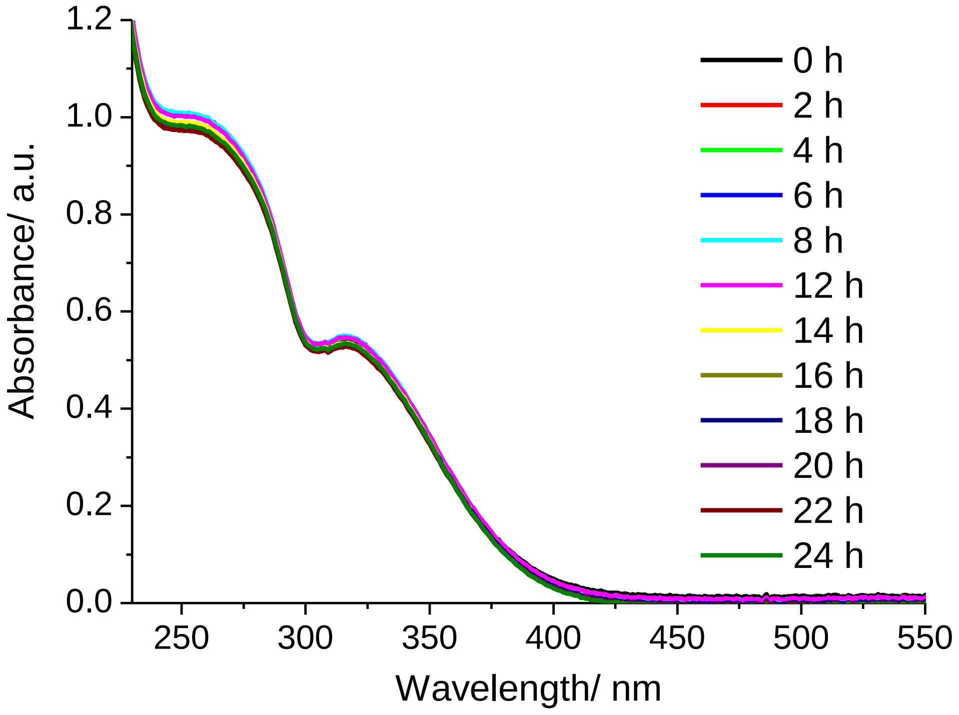

Figure S6. UV-Vis spectrum of **2** (50 μM) in PBS (pH 7.4)/DMSO (200:1) over the course of 24 h at 37 ℃.

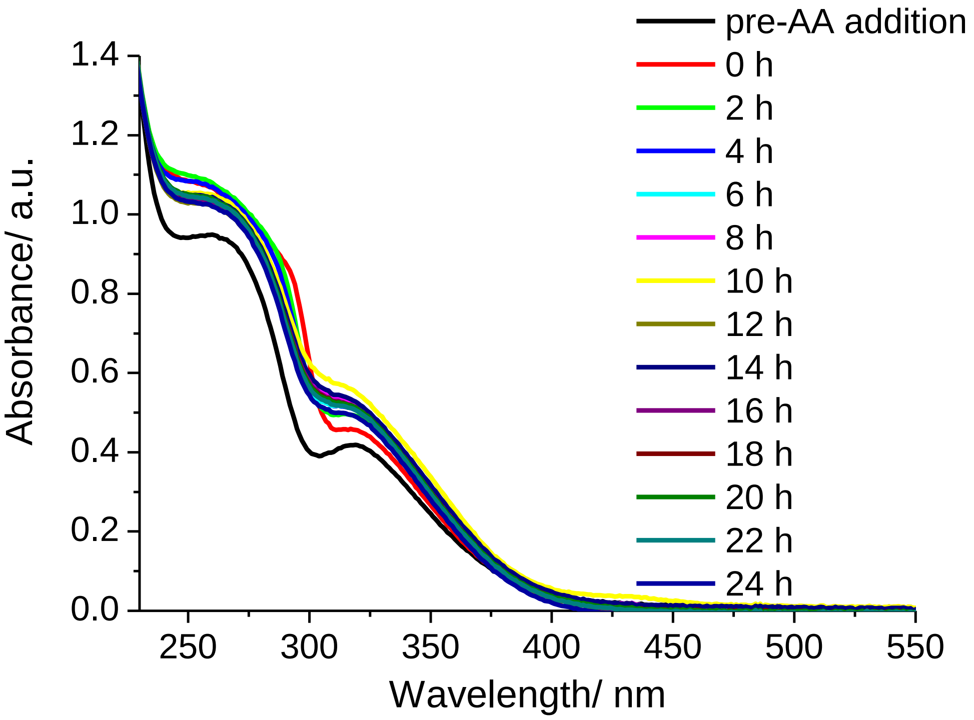

Figure S7. UV-Vis spectrum of **2** (50 μM) in PBS (pH 7.4)/DMSO (200:1) in the presence of ascorbic acid (500 μM) over the course of 24 h at 37 °C.

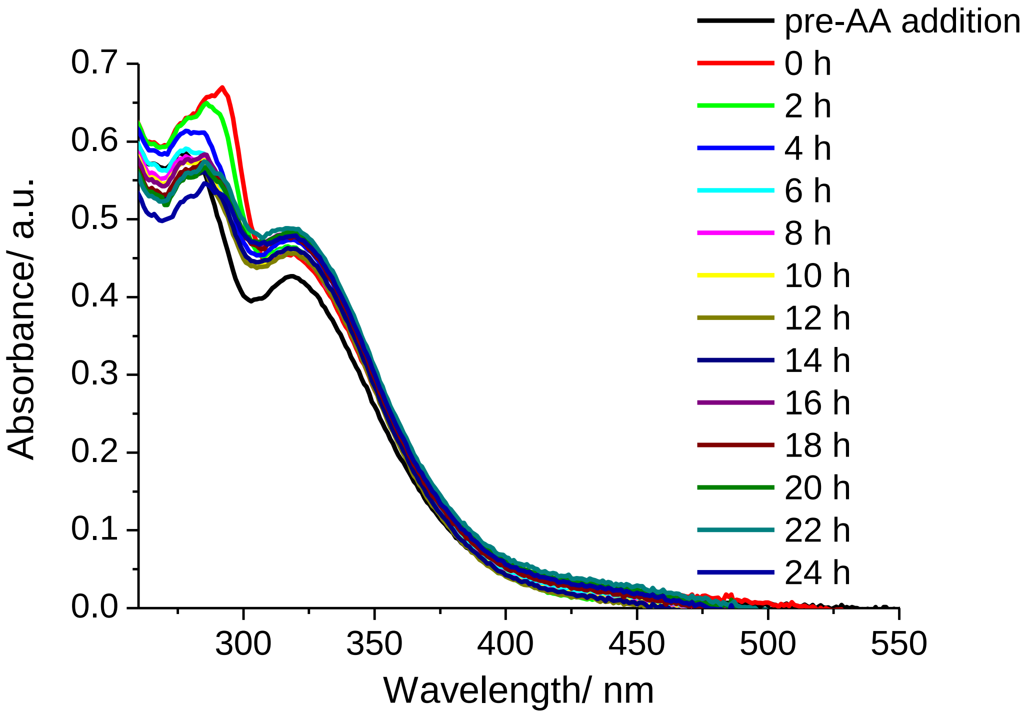

Figure S8. UV-Vis spectrum of **2** (50 μM) in mammary epithelial cell growth medium (MEGM):DMSO (200:1) in the presence of ascorbic acid (500 μM) over the course of 72 h at 37 °C.

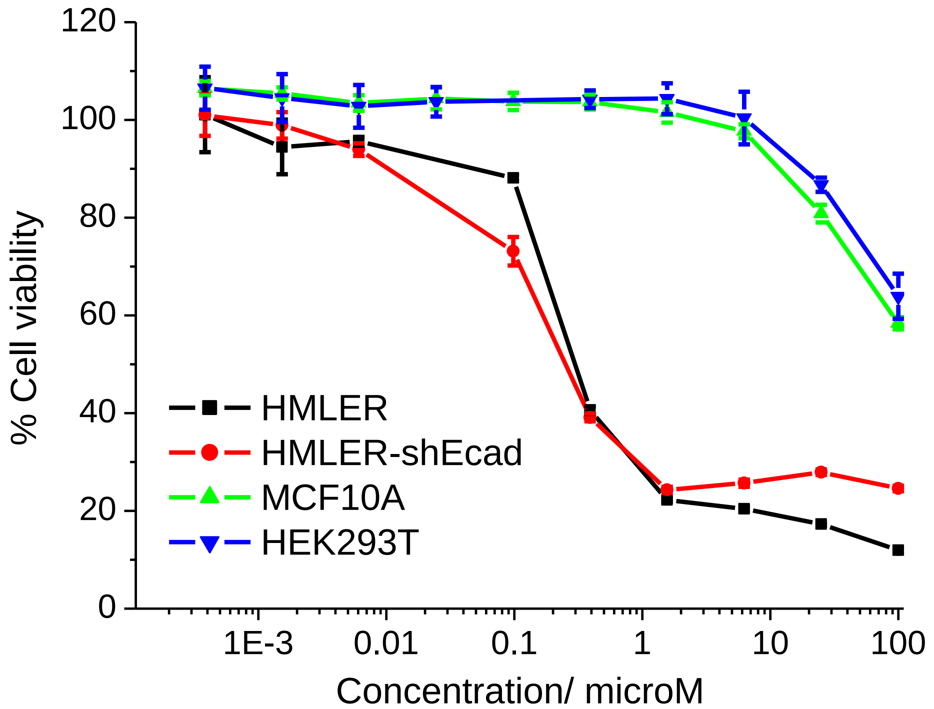

Figure S9. Representative dose-response curves for the treatment of HMLER, HMLER-shEcad, MCF10A, and HEK293T cells with **2** after 72 h incubation.

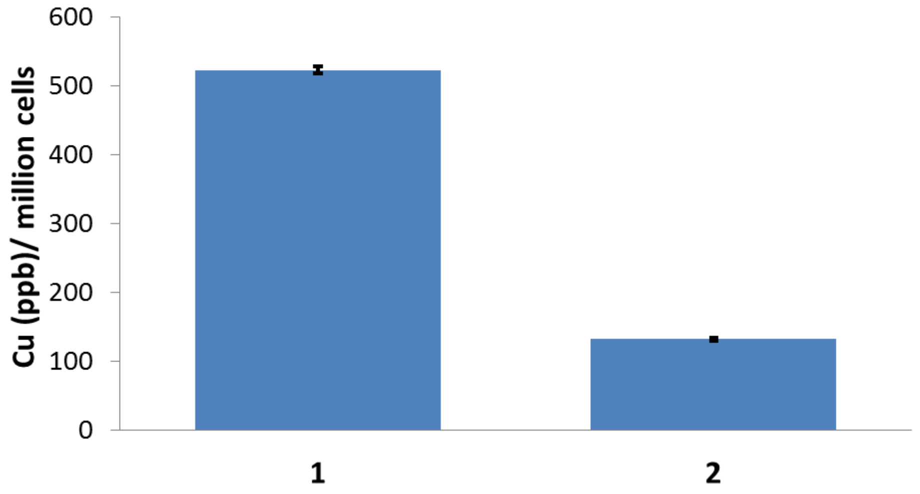

Figure S10. Copper content in whole-cell isolated from HMLER-shEcad cells treated with **1** and **2** (1 µM for 16 h).

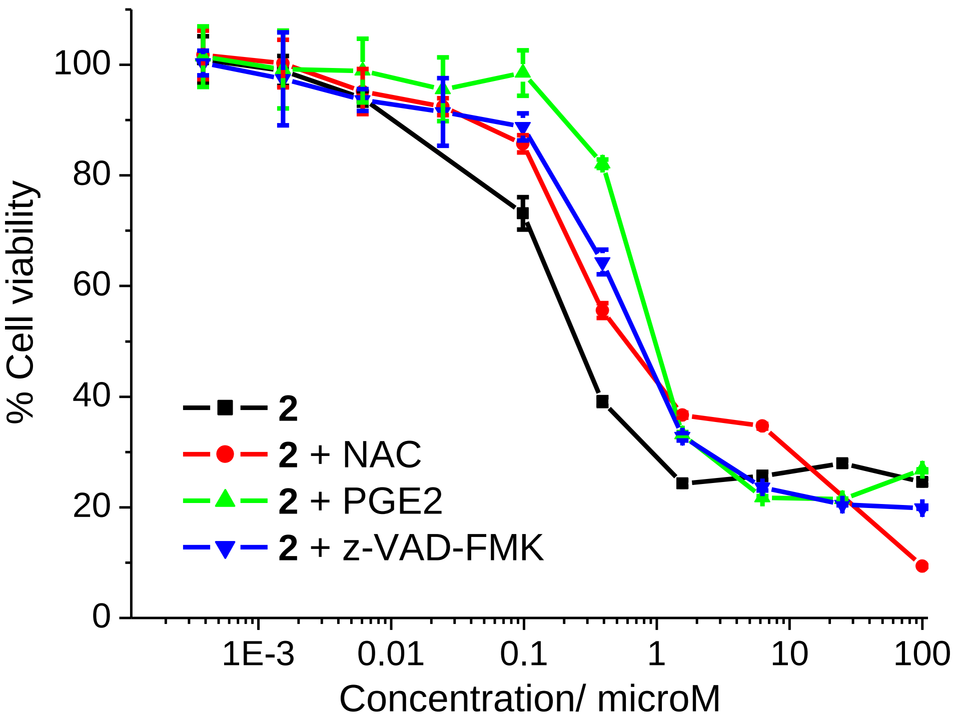

Figure S11. Representative dose response curves of **2** against HMLER-shEcad cells in the absence and presence of *N*-acetylcysteine (2 mM), or PGE2 (20 μM), or z-VAD-FMK (5 μM) after 72 h incubation.
